# Cognitive Dysfunction in the Addictions (CDiA): protocol for a neuron-to-neighbourhood collaborative research program

**DOI:** 10.3389/fpsyt.2025.1455968

**Published:** 2025-05-19

**Authors:** Yuliya S. Nikolova, Anthony C. Ruocco, Daniel Felsky, Shannon Lange, Thomas D. Prevot, Erica Vieira, Daphne Voineskos, Jeffrey D. Wardell, Daniel M. Blumberger, Kevan Clifford, Ravinder Naik Dharavath, Philip Gerretsen, Ahmed N. Hassan, Ingrid M. Hope, Samantha H. Irwin, Sheila K. Jennings, Bernard Le Foll, Osnat Melamed, Josh Orson, Peter Pangarov, Leanne Quigley, Cayley Russell, Kevin Shield, Matthew E. Sloan, Ashley Smoke, Victor Tang, Diana Valdes Cabrera, Wei Wang, Samantha Wells, Rajith Wickramatunga, Etienne Sibille, Lena C. Quilty

**Affiliations:** ^1^ Campbell Family Mental Health Research Institute, Centre for Addiction and Mental Health, Toronto, ON, Canada; ^2^ Department of Psychiatry, University of Toronto, Toronto, ON, Canada; ^3^ Department of Psychological Clinical Science, University of Toronto, Toronto, ON, Canada; ^4^ Department of Psychology, University of Toronto Scarborough, Toronto, ON, Canada; ^5^ Division of Biostatistics, Dalla Lana School of Public Health, University of Toronto, Toronto, ON, Canada; ^6^ Institute of Mental Health Policy Research, Centre for Addiction and Mental Health, Toronto, ON, Canada; ^7^ Institute of Medical Science, Temerty Faculty of Medicine, University of Toronto, Toronto, ON, Canada; ^8^ Department of Pharmacology and Toxicology, Temerty Faculty of Medicine, University of Toronto, Toronto, ON, Canada; ^9^ Department of Psychology, York University, Toronto, ON, Canada; ^10^ Moms Stop the Harm, Victoria, BC, Canada; ^11^ Department of Family and Community Medicine, University of Toronto, Toronto, ON, Canada; ^12^ Ferkauf Graduate School of Psychology, Yeshiva University, New York, NY, United States; ^13^ Ontario Canadian Research Initiative in Substance Matters (CRISM) Node Team, Institute for Mental Health Policy Research, Centre for Addiction and Mental Health, Toronto, ON, Canada; ^14^ Dalla Lana School of Public Health, University of Toronto, Toronto, ON, Canada; ^15^ The Ontario Network of People Who Use Drugs, Toronto, ON, Canada; ^16^ Neuroscience and Mental Health, Hospital for Sick Children, Toronto, ON, Canada; ^17^ Department of Epidemiology and Biostatistics, Western University, London, ON, Canada

**Keywords:** alcohol use disorder, substance use disorder, addiction, cognition, executive function, preclinical, translational

## Abstract

Substance use disorders (SUDs), including Alcohol Use Disorder, are pressing global public health problems. Executive functions (EFs) are prominently featured in mechanistic models of addiction. However, significant gaps remain in our understanding of EFs in SUDs, including the dimensional relationships of EFs to underlying neural circuits, molecular biomarkers, disorder heterogeneity, and functional ability. Transforming health outcomes for people with SUDs requires an integration of clinical, biomedical, preclinical, and health services research. Through such interdisciplinary research, we can develop policies and interventions that align with biopsychosocial models of addiction, addressing the complex cognitive concerns of people with SUDs in a more holistic and effective way. Here, we introduce the design and procedures underlying Cognitive Dysfunction in the Addictions (CDiA), an integrative research program, which aims to fill these knowledge gaps and facilitate research discoveries to enhance treatments for people living with SUDs. The CDiA Program comprises seven interdisciplinary projects that aim to evaluate the central thesis that EF has a crucial role in functional outcomes in SUDs. The projects draw on a diverse sample of adults aged 18-60 (target *N*=400) seeking treatment for SUD, who are followed over one year to identify specific EF domains most associated with improved functioning. Projects 1-3 investigate SUD symptoms, brain circuits, and blood biomarkers and their associations with key EF domains (inhibition, working memory, and set-shifting) and functional outcomes (disability, quality of life). Projects 4 and 5 evaluate interventions for SUDs and their impacts on EF: a clinical trial of repetitive transcranial magnetic stimulation and a preclinical study of potential new pharmacological treatments in rodents. Project 6 links EF to healthcare utilization and is supplemented with a qualitative investigation of EF-related barriers to treatment engagement. Project 7 uses whole-person modeling to integrate the multi-modal data generated across projects, applying clustering and deep learning methods to identify patient subtypes and drive future cross-disciplinary initiatives. The CDiA Program will bring scientific domains together to uncover novel ways in which EFs are linked to SUD severity and functional recovery, and facilitate future discoveries to improve health outcomes in individuals living with SUDs.

## Background

Substance use disorders (SUDs)^1^, including alcohol use disorder (AUD), affect 162 million people worldwide and are associated with substantial morbidity, mortality, and disability (1–4). The socioeconomic and health impacts associated with the use of alcohol, cannabis, and illicit drugs, substantially contribute to the global burden in years living with a disability and lost to premature death (5, 6). In 2020, the World Health Organization heightened their calls for enhanced actions to curb the harmful effects of alcohol use, underscoring the global public health priority of the damaging consequences of alcohol use (7). In the US, 140,000 people die annually from excessive alcohol consumption (i.e., consuming more than 25 grams of ethanol per day) and another 70,000 from drug-related causes, costing the U.S. economy 191.6 billion dollars related to alcohol and 151.4 billion dollars due to use of other substances (8–10). Similarly, in Canada, alcohol and drug use causes almost 20,000 deaths, and costs the Canadian economy nearly 40 billion dollars annually (3). Treatments for SUDs have evidence supporting their efficacy but their availability is limited, highlighting the critical need to increase access to treatments and develop novel and improved interventions for SUD (11).

To advance the understanding of addiction, research on the cognitive and neurobiological factors involved in the development and maintenance of SUD is crucial (12). Contemporary models underscore the centrality of cognition to different phases of SUD development, with a strong emphasis on executive functions (EF) such as response inhibition and decision-making, in conjunction with motivational (e.g., incentive salience, cue reactivity/craving) and affective (e.g., negative emotionality) factors (13, 14). While motivational and affective factors have received extensive attention in the field of SUD research [e.g (15–19)], comprehensive studies of EF and its component processes are relatively scarce, despite the proposed centrality of EF to treatment response and functional outcomes (20, 21).

Further, studies to date have most commonly used relatively “pure” or homogeneous patient groups (e.g., adults with a specific SUD and with no psychiatric comorbidities), with more limited attention to individual differences or within-group variability (22–24). Yet, adults seeking treatment for addiction represent a highly heterogeneous group, with complex substance use histories, often meeting criteria for multiple SUDs, and a high prevalence of psychiatric comorbidities (25–27). Since “pure group” studies have limited generalizability to patient populations seen in real-world clinical settings, there is a critical need to characterize heterogeneity of executive dysfunction in a large inclusive cohort study involving a complex patient population that is characteristic of large tertiary care facilities.

To fill these gaps, we introduce the protocol for the Cognitive Dysfunction in the Addictions (CDiA) program - an integrative team-science and translational research program aiming to evaluate the central thesis that EF is crucial to SUD pathophysiology and functional outcomes. The program aims to aid the discovery of shared versus uniquely affected EF domains (28, 29) across 7 interconnected projects spanning a diverse set of methodologies and perspectives.

While there is ongoing debate about the precise number of “core” processes that define EF, converging evidence suggests that EF comprises at least three factors that are related but distinct on a behavioral and neurobiological level (30–34): 1) *inhibition*, or the ability to prevent the processing of irrelevant information in working memory and/or inhibit a context-inappropriate behavioral response; 2) *working memory updating*, or the ability to monitor the contents of working memory for relevance to the current task and to remove from or add information to working memory; and 3) *set shifting*, reflecting the ability to switch between multiple operations or task sets. These processes are supported by common and dissociable neural substrates within a distributed network of frontoparietal brain regions (35), subserved by known biological and neurotransmitter systems (36, 37), and may be partially genetically influenced (38). EF “deficits” refer to impaired functioning in one or more of these domains, whereas EF “biases” reflect the specific or selective influences of motivational or emotional contexts or materials on EF performance (39).

SUDs can be associated with both deficits and biases in EF accompanied by structural and functional alterations in underlying neural circuitry (40). Problems with EF lead to functional impairment across all stages of addiction. Interventions that bolster EF may mitigate the influence of attentional and behavioral control on drug-seeking and use. For example, computerized cognitive retraining may improve working memory capacity (41–43) and reduce impulsive choice and valuation of specific rewards in addiction (44). Further, non-invasive brain stimulation has exhibited promise across a range of SUDs and appears to impact both craving and decision-making processes (45–47). However, the deficits and biases of EF most linked to functional outcomes, as well as their respective biological bases, remain to be identified and targeted for maximal therapeutic benefit.

By embedding a multilevel assessment of EF in an interdisciplinary research program, CDiA will aid the discovery of shared versus uniquely affected EF domains (28, 29) in a large heterogeneous cohort of adults seeking help for SUDs. Parallel biomarker studies in human participants and translational mechanistic studies in relevant preclinical models will link domains of EF to putative biological underpinnings, together paving the way for the rational design of targeted and individualized therapeutics for treatment and improving functional outcomes in SUDs. Linkages to health care administrative databases will further mobilize knowledge to help shape public health policy and effect change at the societal level.

CDiA consists of seven interconnected projects (P1-7) with the following main objectives, each designed to evaluate and elaborate on the Program’s central tenet that EF is crucial to SUD functional outcomes:

1. To identify domains of EF linked to functional outcomes in adults seeking treatment for SUD (Project 1).
2. To identify imaging biomarkers (MRI, fMRI) associated with domains of EF and those most predictive of functional outcomes in adult outpatients seeking treatment for SUD (Project 2).
3. To identify biomarkers mechanistically associated with EF and functional outcomes in adult outpatients seeking treatment for SUD (Project 3).
4. To assess the impact of repetitive transcranial magnetic stimulation (rTMS) on EF deficits in adult outpatients seeking treatment for comorbid AUD and major depressive disorder (Project 4).
5. To study the impact of alcohol on EF deficits, and their reversal by novel therapeutic interventions, in a preclinical animal model (Project 5).
6. To assess links between EF, treatment-seeking for SUD, and healthcare utilization and costs (Project 6).
7. To identify subtypes of individuals seeking SUD treatment using cross-disciplinary data types from all projects and map the biopsychosocial drivers of cognitive dysfunction in SUDs (Project 7).

Apart for the preclinical project (Project 5), all projects draw on a common sample of adults seeking treatment for SUD, allowing for the triangulation of evidence within the same pool of participants. Alcohol was chosen as the main substance of interest in Projects 4-5 and the qualitative component of Project 6, as those are targeted experimental/qualitative studies that necessitate smaller sample sizes with more homogeneous characteristics. This choice was also dictated by epidemiological estimates, which suggest AUD is the most common type of SUD observed in the US and Canada (48, 49). AUD is therefore also expected to be the most common diagnosis in CDiA. Once established, the proposed research platform can be used to study other types of SUD or more complex populations in the future.

The CDiA Program benefits from the full participation of a Living Expertise Research Advisory (LERA) committee, which is composed of community members who self-identify as having living or lived experience with SUD either directly or as a family member. Incorporating the perspectives of individuals with lived/living experience helps to increase the relevance and impacts of the research on the communities expected to benefit from the findings (50). In conjunction with the scientists leading the program, the LERA committee contributed to the formulation of the program and has offered ongoing insights into its public-facing materials and study procedures. The LERA committee will play a central role in interpreting the CDiA findings and sharing the results with knowledge users in the community. The CDiA Program also consults with an International Scientific Advisory Committee composed of SUD experts who provide ongoing guidance.

**Figure 1:**
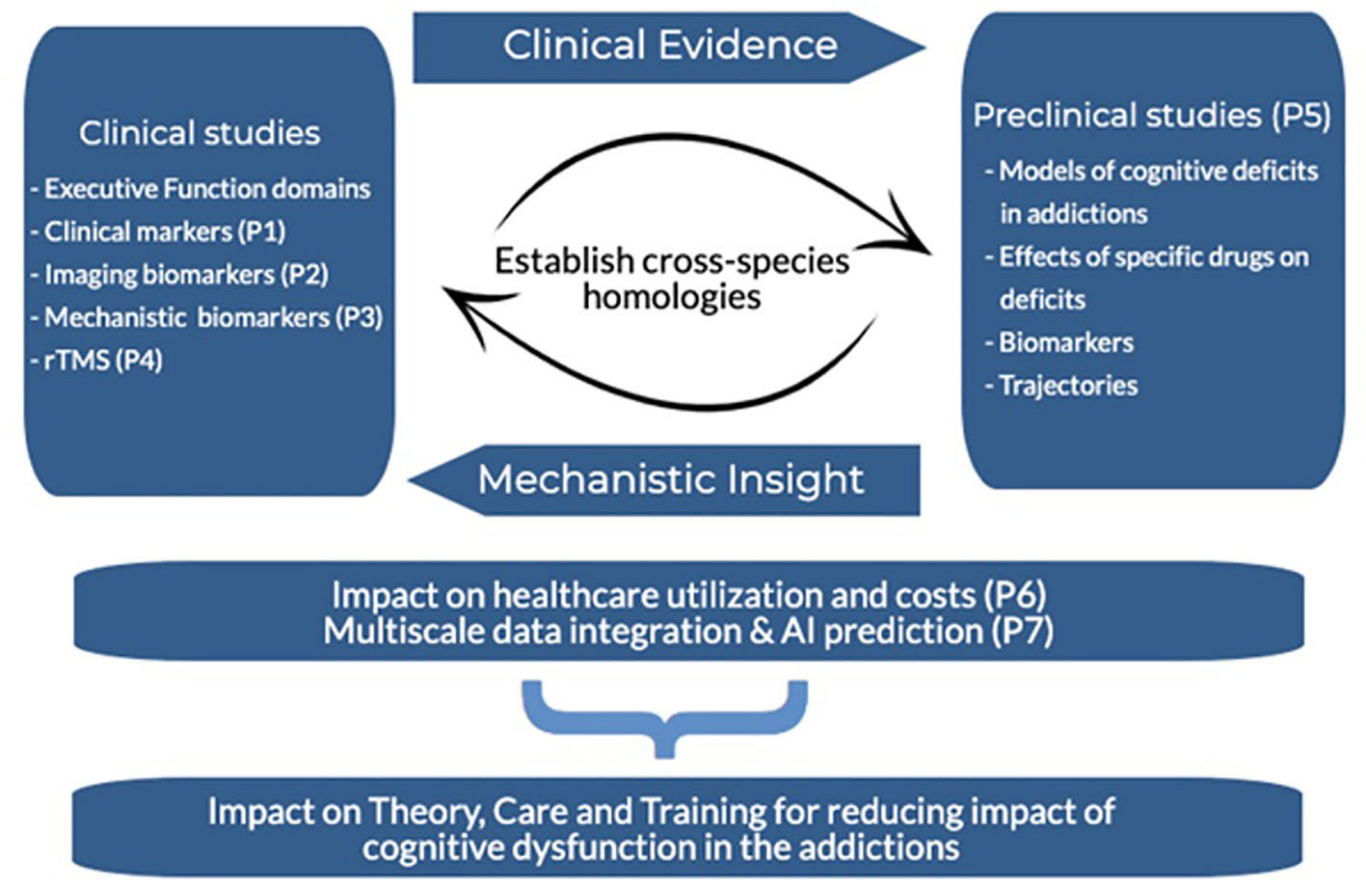
Overview of the CDiA research program.

An overview of the CDiA Program and synergy between Projects is presented in **Figure 1**. A detailed description of each project, including the background, methods, and analytical approach, is presented below.

## Project 1: to identify domains of EF linked to functional outcomes in adults seeking treatment for SUD

### Background

Dual Process models of addiction suggest that SUDs can result from an imbalance between a controlled process that is more intentional (for which EF are central), and an automatic process that is more implicit or involuntary (for which affective states are influential) (51, 52). EFs modulating either appetitive (desirable) or aversive (unpleasant) motivational states are compromised in those with SUDs ( (53); also see the Addiction Neuroclinical Assessment [13, 54, 55)]. Although many studies have investigated how deficits in EFs relate to outcomes among people with SUDs (56), the existing studies are often limited by small samples, very brief (3-6 month) follow up periods, narrow focus on a single substance, the absence of mental health comorbidities, and limited attention to functional outcomes (56). Further, most studies examine either EF deficits (i.e., general impairments in EF performance) or biases (i.e., EF impairments in the context of emotional or motivational material), without examining their relative importance for SUD outcomes. Thus, there is a need for integrative research incorporating all of these factors, and carefully evaluating links with both clinical and functional outcomes in a heterogeneous clinical sample followed prospectively over time.

### Objectives

The primary goals of Project 1 (P1) are to characterize EF deficits and biases and to determine which domains of EF are most predictive of functional and treatment outcomes in a complex population of adults seeking treatment for SUD to test the following hypotheses (H1a-e):

#### H1a

Deficits and biases in the three EF domains will exhibit stronger baseline associations with indicators of functioning, such as disability and quality of life, relative to other components of cognitive functioning.

#### H1b

EF deficits across all three domains will be most prominent in those with greater (i) severity and (ii) duration of SUD, whereas EF biases will be most prominent in those with concurrent mood disorders and elevated suicidality.

#### H1c

EF deficits and biases can be used to identify clusters of participants that are associated with distinct clinical features.

#### H1d

Relatively more severe EF deficits and biases at baseline will predict slower improvements in functional outcomes over and above substance use, treatment engagement, and healthcare utilization.

#### H1e

EF domains will show improvements from baseline to the one-year follow-up. Improvements in EF will be positively associated with improvements in SUD symptoms and in functional outcomes.

### Methods and planned analyses

#### Design and participants

The CDiA Program aims to recruit a total sample of 400 adults seeking treatment for AUD and/or SUD to participate in this observational, longitudinal study involving clinical and cognitive assessments at baseline and at a one-year follow-up period (see **Table 1** for a comprehensive list of clinical and cognitive assessments and the time points of each assessment). Inclusion criteria are: (1) 18 - 60 years of age; (2) meeting diagnostic criteria for a current (past year) AUD and/or SUD (not counting nicotine- or caffeine-related disorders); and (3) seeking support for substance use concerns. Exclusion criteria include: (1) acute intoxication or withdrawal; (2) active psychosis; (3) acute suicidality; and (4) history of severe head injury, dementia, severe neurodevelopmental disorders, and other medical conditions or medications that could severely impair cognition.

**Table 1:** Schedule of clinical & cognitive assessments.

Procedure	Construct Measured	Baseline ^a^	Follow up ^b^	Year End ^c^
Informed Consent	N/A	✓		
Verbal confirmation of 12-hour abstinence and time of last substance use	Substance Use	✓		✓
Urinalysis and Breathalyzer	Substance Use	✓		✓
Performance-Based Measures				
CNS-Vitals ^¢ θ^	Cognitive Functioning: General	✓		✓
Iowa Gambling Task ^¢ θ^	Cognitive Functioning: Decision Making	✓		✓
Delay Discounting Task ^¢^	Cognitive Functioning: Decision Making	✓		✓
Probability Discounting Task ^¢^	Cognitive Functioning: Decision Making	✓		✓
Flanker Task ^¢ θ^	Executive Functioning: Inhibitory Control	✓		✓
N-back Task ^¢ θ^	Executive Functioning: Updating	✓		✓
Switch Task ^¢ θ^	Executive Functioning: Set Shifting	✓		✓
Interview-Based Measures				
Diagnostic Assessment Research Tool (55)^d^	AUD, SUD, and other mental health diagnoses	✓		✓
Timeline Follow Back (56)	Substance Use	✓		✓
Psychiatric Treatment History	Treatment History: Medications	✓		✓
Psychological Treatment	Treatment History: Psychological	✓		✓
Self-Report Measures				
Demographics ^$^	Identity	✓		
Bem Sex Role Inventory (Short Form) (68, 69) ^$^	Identity	✓		
Lifetime substance use history (70) ^$^	Substance Use	✓		
Alcohol Use Disorders Identification Test (71) ^$^	Substance Use and Harms	✓		✓
Drug Use Disorders Identification Test (72) ^$^	Substance Use and Harms	✓		✓
Fagerström Test for Nicotine Dependence (73) ^$^	Substance Use and Harms	✓		✓
Alcohol Use Awareness and Insight Scale (74) ^$^	Substance Use Awareness	✓		✓
Substance Use Awareness and Insight Scale (75) ^$^	Substance Use Awareness	✓		✓
Severity of Dependence Scale (76) ^$^	Substance Use and Harms	✓	✓	✓
Brief Substance Craving Scale (77) ^$^	Substance Use: Craving	✓		✓
Stages of Change Readiness and Treatment Eagerness Scale (78) ^$^	Substance Use Goals	✓		✓
Substance Use Motives Measure (79) ^$^	Substance Use: Motivation	✓		✓
WHO Disability Assessment Schedule 2.0 (80) ^$^	Functional Outcomes	✓	✓	✓
WHO Quality of life - Brief (81) ^$^	Functional Outcomes	✓	✓	✓
Patient Health Questionnaire - 9 (82) ^$^	Clinical Features: Depression	✓		✓
Generalized Anxiety Disorder - 7 (83) ^$^	Clinical Features: Anxiety	✓		✓
Childhood Trauma Questionnaire (84) ^$^	Clinical Features: Risk Factors	✓		
List of Threatening Experiences Questionnaire (85) ^$^	Clinical Features: Risk Factors	✓		✓
Coping Orientation to Problems Experienced Inventory, abbreviated (86) ^$^	Clinical Features: Risk Factors	✓		✓
Difficulties in Emotion Regulation Scale -Short Form (87) ^$^	Clinical Features: Risk Factors	✓		✓
PTSD Checklist for DSM-5 (88) ^$^	Clinical Features: PTSD	✓		✓
Wender Utah Rating Scale (89) ^$^	Clinical Features: ADHD	✓		✓
Personality Inventory for DSM – Brief Form (PID-5-BF+) (90) ^$^	Clinical Features: Personality	✓		✓
UPPS-P Impulsivity Scales - Short Form (91) ^$^	Clinical Features: Personality	✓		✓

Check marks indicate the time point at which each measure is administered.^$^self-administered with aid of research personnel.

^¢^computer-based tasks.

^θ^mandatory onsite administration.

a post-informed consent, two sessions within 1 month (360 min).

b bimonthly at months 2, 4, 6, 8, and 10, +/- 1 month; (45 min).

c two sessions to be administered at month-12, +/- 1 month (360 min).

d DART subset to assess presence/absence of the following DSM-5-TR diagnoses: Substance-related and addictive disorders (alcohol use disorder, gambling disorder, substance use disorders); Depressive disorders (major depressive disorder, persistent depressive disorder); Bipolar and related disorders (hypomanic or manic episodes, bipolar I disorder, bipolar II disorder, cyclothymic disorder); Anxiety disorders (panic disorder, agoraphobia, generalized anxiety disorder, social anxiety disorder, specific phobia); Obsessive-compulsive and related disorders (obsessive-compulsive disorder); Trauma-and-stressor related disorders (posttraumatic stress disorder); Feeding and eating disorders (anorexia nervosa, avoidant/restrictive food intake disorder, bulimia nervosa, binge eating disorder); Schizophrenia spectrum and related psychotic disorders (delusional disorder, brief psychotic disorder, schizophreniform disorder, schizophrenia, schizoaffective disorder, substance-induced psychotic disorder).

#### Measures

Interview measures will include the Diagnostic Assessment Research Tool (DART (57) to assess SUD and comorbid diagnoses, and the Timeline Follow-Back (TFLB (58) to characterize alcohol and substance use over the past 60 days. Self-report items assessing lifetime history of alcohol and substance use will also be administered, along with measures of severity of alcohol- and substance-related harms (see **Table 1**). We will measure both (a) general EF deficits and (b) biases in these functions associated with emotional and motivational states; both neutral and emotional stimuli are used in our EF tasks. The stimuli for the EF tasks are a pool of happy, neutral, and sad faces from the Karolinska Emotional Directed Faces database (KEDF (59). Each task is administered with trial blocks with emotional stimuli (i.e., to measure EF biases) and trial blocks with neutral stimuli (i.e., to measure EF deficits). A flanker task (60–63) will be used to assess inhibition, an n-back task (64, 65) will be used to assess working memory updating, and a switch task (66) will be used to assess set-shifting.

Global cognitive function will be evaluated with the Central Nervous System Vital Signs (CNS-VS (67) computerized assessment, which has been validated for use in addiction. To minimize the impact of acute intoxication on cognition and EF, we will verbally confirm participants have not used alcohol/substances 12 hours prior to their cognitive assessment (Last Drug Use Verification Form) and administer a breathalyzer test to confirm zero blood alcohol concentration at the time of testing. We will also administer a urine drug screen and collect self-report data on time since last use of each drug to account for recency of use in the analyses. Other factors relevant to understanding substance use patterns are also assessed, including demographic factors, readiness to change, substance use motives, SUD insight, SUD treatment history, and concurrent mental health difficulties. The complete battery of measures is compiled to provide a fulsome characterization of the sample, consistent with the unique perspectives of each CDiA project and with the intended generativity of the CDiA Program for future research (see **Table 1** for a full list of measures).

### Power analysis and data analytic plan

Multiple regression will be used with the clinical, functional, and demographic factors entered as simultaneous predictors of each EF domain to test hypotheses about which factors are most strongly associated with specific EF domains (H1a and H1b).

For H1c, we will conduct latent profile analyses using domains of EF as indicators of discrete latent profiles of relative deficits and biases. We will then examine the prediction of membership in these latent profiles from substance use profiles.

For H1d, latent growth curve modeling will be used to model changes in substance use frequency, SUD severity, and functional outcomes over the follow up time points. Next, rate of change will be examined as a function of baseline EF (H1a) by regressing variability in the slopes for the growth factors on baseline EF deficits and biases. These will be included as simultaneous predictors in the model to examine the specific EF domains and biases that are most predictive of change in the clinical and functional outcomes.

For H1e, we will use a generalized linear mixed effects modelling approach. EF deficits and biases will be modelled as dependent variables and time will be entered as a within-subjects factor to determine if there is significant within-person change from baseline to one year follow up. Changes over time in SUD symptoms and functional outcomes will be modelled using the same approach, with changes in EFs included in the models as a time-varying predictor of changes in these outcomes.

## Project 2: to identify neuroimaging biomarkers associated with domains of EF and SUD symptoms most predictive of functional outcomes

### Background

Whereas Project 1 (P1) will identify EF deficits that may be potential targets of cognitive interventions in future work, Project 2 (P2) will identify neuroimaging biomarkers associated with the same core EF domains and functional outcomes evaluated in P1 to inform innovative brain-based treatments for SUDs. To do so, a complementary mechanistic understanding of neural system dysfunctions that underlie behavioral EF deficits is needed.

Functional magnetic resonance imaging (fMRI) studies in healthy adults suggest that the three dimensions of EF have both common and distinct neural substrates. Studies investigating the three EF domains side by side show overlapping activation in frontoparietal regions, most notably the inferior frontal gyrus, anterior cingulate cortex and bilateral parietal cortices (92). The ability to maintain a task goal stably, shared across all EF domains, in turn relies on areas of the lateral prefrontal cortex (PFC) (30, 93, 94), anterior cingulate cortex (ACC) and frontal operculum (95).

In contrast, inhibition is associated with more extensive recruitment of temporal regions and the cortico-striatal circuit (96). Set-shifting may uniquely involve more posterior dlPFC regions and more extensive parietal areas (97, 98). Working memory updating has been additionally mapped onto fronto-striatal connections and requires input from the basal ganglia and cerebellum (99–101). Similar partially dissociable signatures of the three distinct EF dimensions have been identified in studies using resting-state (i.e., task-free) fMRI (102, 103), structural MRI (104, 105), and diffusion-weighted imaging (DWI) (104).

Meta-analyses of task-based fMRI across SUDs show altered activation in frontoparietal and striatal regions across studies (106). Importantly, however, prior studies have almost universally focused on a single EF domain, providing relatively limited insight into the complex cognitive architecture that may underlie EF impairment in SUDs (107–109). Clinically relevant insight is further limited by a predominance of cross-sectional studies, which do not investigate how neural features supporting EF may contribute to symptom change and functional recovery over time. Characterizing executive dysfunction heterogeneities and underlying neural circuits longitudinally across large naturalistic cohorts of patients seeking treatment for SUDs is likely to provide novel insight into the pathophysiology of addiction and help identify novel targets for brain-based interventions.

### Objectives

The first objective of P2 is to identify novel imaging biomarkers (MRI, fMRI) uniquely associated with each of the three domains of EF in SUDs. Its second objective is to identify relationships between these neurostructural and functional markers and SUD severity and day-to-day functioning, both cross-sectionally and over a one-year follow-up. These imaging biomarkers may help guide future development and individualization of biologically informed treatments for SUDs, e.g., through neuromodulation (see P4) or pharmacotherapies (see P5).

#### H2a

Behavioral performance on the three EF domains will map onto partially dissociable neural substrates overlapping with frontoparietal and corticostriatal circuits. The extent of activation and pattern of brain activity associated with each of the three EFs will be associated with greater SUD severity and lower general functioning at baseline.

#### H2b

Greater disturbances in EF-associated neural circuits at baseline will be associated with lesser improvement in SUD severity and functional outcomes on follow-up, above and beyond impairments in other neural circuits.

### Methods and planned analyses

#### Participants

All participants included in the total sample are invited to participate in a 1.5-hour MRI protocol. P2-specific exclusion criteria include the presence of MRI-incompatible metal implants, history of stroke, and claustrophobia. We anticipate approximately 50% of the entire sample (n=200) will be eligible and consent to MRI. Eligible participants are invited for a longitudinal scan at one-year follow-up; we anticipate 25% (n=50) being available and consenting to the repeat scan to assess longitudinal stability of our main measures. Given the specific safety requirements of MRI, it is possible that the final P2 sample will not be fully representative of the P1 sample in terms of demographics and clinical characteristics. If this is the case, we will consider this important caveat in interpreting any results using joint P1-P2 and conduct sensitivity analyses where appropriate.

#### MRI methods

Our research imaging protocol uses state-of-the-art imaging sequences fully harmonized with MRI acquisition protocols used by large-scale population-representative studies (110) and other ongoing CAMH-based cohort studies (111, 112) (see **Table 2**), which will allow us to leverage our data for comparison or transdiagnostic analyses. To support new research studies, a portion of the scanning time is secured for pilot sequences.

**Table 2:** MRI sequences.

Whole-brain MRI Sequence	Duration (minutes)	Measure	Technical Parameters
3D T1 Magnetization Prepared RApid Gradient Echo (MPRAGE)	6:11	Total, interhemispheric, and/or regional cortical thickness (mm), cortical surface area (mm^2^), total and regional brain volume (mm^3^)	Field of view (FOV) 256 x 256 mm^2^ TR = 6.952 ms TE = 2.920 ms TI = 1060 ms 1 mm isotropic voxel size Parallel imaging factor ARC = 2 Slices per 3D slab = 208
Axial resting state functional MRI (fMRI)	10:00	Resting-state connectivity	FOV 216 x 216 mm^2^ TR = 800 ms TE = 30 ms Number of slices = 60 (no gap) Flip angle = 52° Matrix 90 x 90 2.4 mm isotropic voxel size Hyperband multi-slice acceleration factor = 6 Run time points 750
Axial N-Back fMRI	9:40	Executive Functioning: working memory	Same parameters as before Run time 725
Axial GoNoGo fMRI	7:16	Executive Functioning: inhibition	545
Axial Letter Judgement Task fMRI	7:36	Executive Functioning: set-shifting	570
Axial Diffusion MRI	7:11	Brain tissue microstructure	FOV 240 x 240 mm^2^ TR = 4100 ms TE = 81.7 ms Number of slices = 81 (no gap) Matrix 140 x 140 b-values (directions): b0 (8), b=500 s/mm^2^ (6), b=1000 s/mm^2^ (15), b=2000 s/mm^2^ (15), b=3000 s/mm^2^ (30) Hyperband multi-slice acceleration factor = 3
Oblique Axial Neuromelanin GRE	8:01	NeuroMelanin Sensitive MRI	Partial brain coverage with FOV = 165 x 220 mm^2^ TR = 284 ms TE = 4.1 ms Number of slices = 10 Slice thickness = 2.5 mm Slice gap = 0 mm Flip angle = 50° Matrix = 320 × 512

##### Structural measures

A standard T1-weighted structural scan is acquired to obtain detailed measures of cortical morphology (e.g., thickness, surface area, and volume). Diffusion-weighted imaging (DWI) is acquired to measure white matter tract integrity and tissue microstructure. Neuromelanin-sensitive (NM-MRI) scans are acquired to quantify the integrity of the substantia nigra (113, 114), with partial coverage of the locus coeruleus (115). NM-MRI scans were included to capture metrics related to catecholamine (i.e., dopamine and norepinephrine) signaling implicated in EF (116), and SUDs (113, 117), based on molecular imaging and preclinical models (118). NM-MRI captures the integrity of brain nuclei and neuromodulatory systems that are relatively conserved across species (119, 120). Thus, it also represents a unique bridge to the preclinical component of CDiA as described in Project 5 below.

##### Resting-state BOLD

Participants are asked to remain awake with their eyes open for 10 minutes while lying restfully in the scanner.

##### Cognitive fMRI paradigms

**Figure 2:**
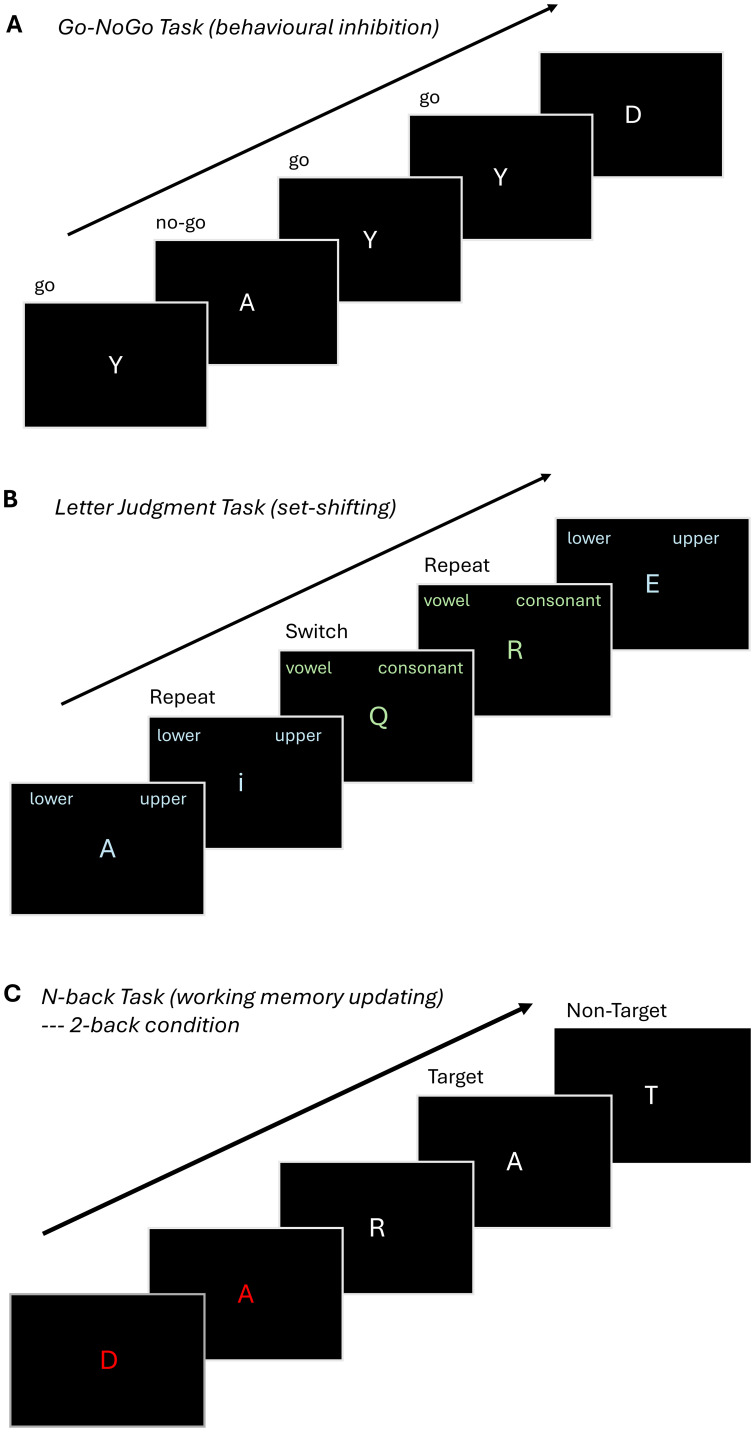
Overview of fMRI tasks. **(A)** Go-NoGo: Participants are shown a series of letters, one at a time, on a monitor, and are instructed to respond when they see the letter 'Y' (Go Condition) and not to respond (No Go Condition) to other letters. The task comprises two blocks with 90 Go Conditions and 30 No Go Conditions (75% Go and 25% No Go). **(B)** .Letter Judgement Task: Participants view one of two possible conditions and are instructed to indicate whether a letter presented on the screen is uppercase or lowercase, or whether it is a vowel or a consonant, respectively. The task comprises a single block with 30 switch trials, where the instructions on the current trial are different from the preceeding one and 30 repeat trials in which the instructions on the current trial match the preceeding one. **(C)** .N-back:The participant is shown a sequence of letters and is instructed to respond when the letter on the monitor matches the letter presented 2 trials earlier (2-back condition). The tasks consists of three blocks of the 2-back condition and three blocks of a 0-back condition, where participants are instructed to press a button when the letter X is presented (not visualized).

We administer three EF tasks for a total duration of ~30 minutes. The tasks assess the same EF domains as in P1 but were specifically selected to be distinct from the paradigms used in P1 to reduce practice effects, which may alter in-scanner performance and brain activation patterns (121, 122). The EF cognitive paradigms were adapted from Rieck et al. (92) and are presented in a pseudorandom order counterbalanced across participants. To minimize visual distraction and permit joint analysis across all EF dimensions, the tasks were designed to be visually simple and consistent across EF domains, using white or light-colored letter stimuli against a dark grey background (**Figure 2**).

##### Proposed analysis

At baseline, task-based brain activation and brain structure will be investigated in association with SUD symptom severity, functional ability and out-of-scanner EF performance as measured by P1. Multivariate approaches (e.g., Partial Least Squares regression) will be used to investigate relationships between brain structure, function, and performance across the three EF tasks by identifying latent variables (LV) that capture covariance between the set of predictors (microstructure, performance) and outcome variables (functional activation). Individual brain scores reflecting the degree to which participants expressed the LV activation pattern will be correlated with SUD severity, functional ability and out-of-scanner EF performance, at baseline and on follow-up, uncovering neural factors crucial for improved functional outcomes. Age, sex, and primary substance of concern will be used as covariates. We anticipate the EF-domain-specificity of the neuroimaging results will mirror patterns obtained using out-of-scanner EF measures in P1. Although it is challenging to provide a power estimate for studies involving complex multivariate analytic approaches in neuroimaging, our proposed sample size is consistent with recent empirically derived recommendations to enhance reproducibility in task-based fMRI research (123). These studies suggest that a sample size of n=120 is sufficient to obtain a test-retest voxel-wise correlation >0.80 across most paradigms. Thus, our proposed cross-sectional sample size of n=200, is in line with these recommendations. Further, based on standard power analysis tools, a sample size of 200 allows power of >0.80 to detect small effect sizes on the order of r=0.2 or Cohen’s d=0.2.

## Project 3: to identify biomarkers mechanistically associated with EF and functional outcomes in adult outpatients seeking treatment for SUD

### Background

Identifying neural biomarkers associated with EF in SUDs (P2) will provide valuable insight into neural mechanisms that may be targeted via brain-based therapies. However, imaging is relatively costly, not accessible to everyone (124, 125), and may offer limited insight into molecular mechanisms that may be targetable through pharmacological interventions. To address these limitations, Project 3 (P3) will assess peripheral blood-based biomarkers and provide insight into the molecular mechanisms underlying EF deficits in SUDs. The analysis of peripheral biomarkers has indeed emerged as a promising approach to probe potential mechanisms or inform prognosis for SUDs (126, 127). Establishing the relationship between peripheral biomarkers and cognitive dysfunction in SUDs can help to unravel contributing mechanisms, including domains of EF, and to understand associated risk and mediating factors.

**Figure 3:**
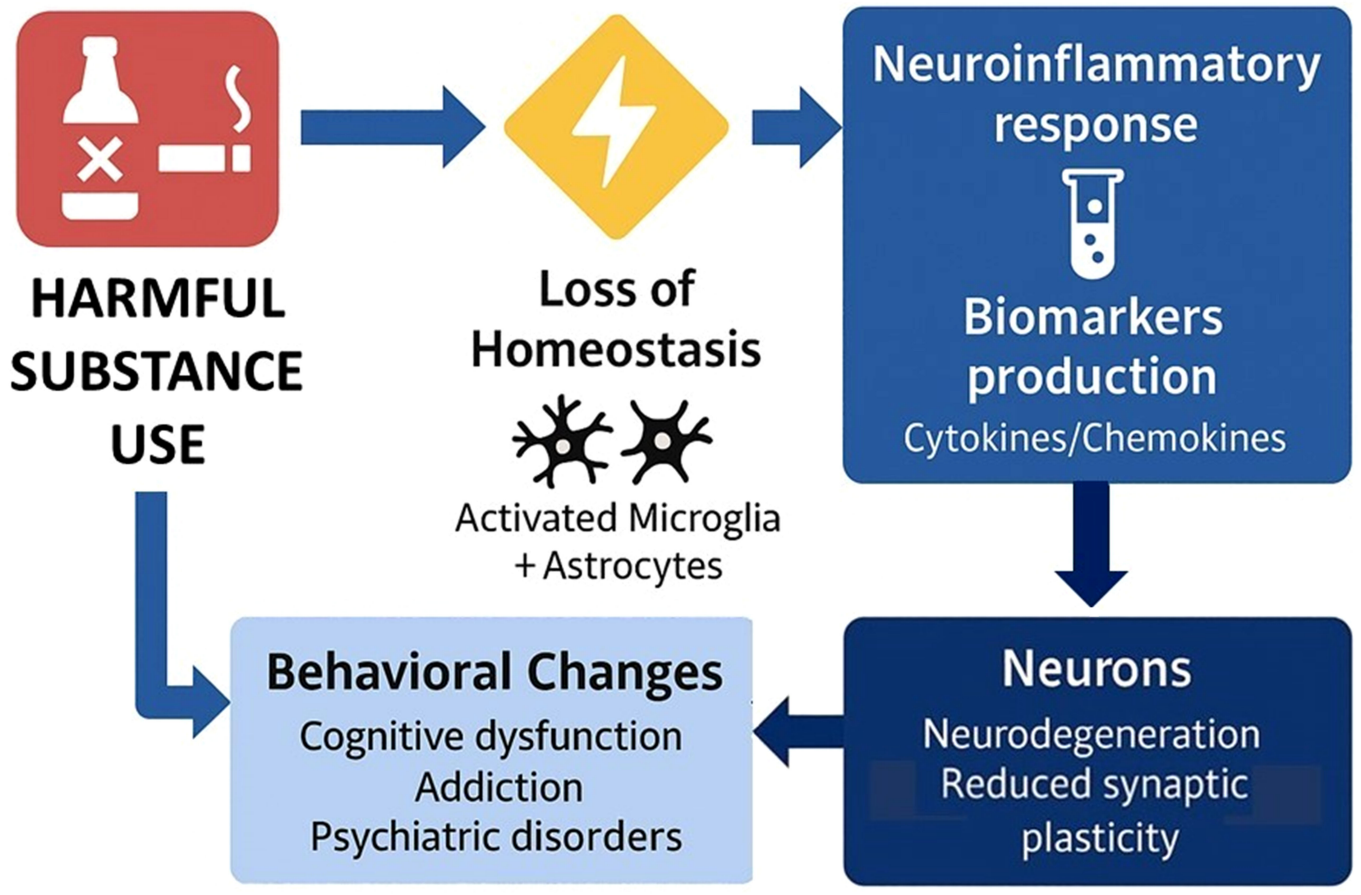
Harmful substance use induces activation of glia cells and neuroinflammation.

Substance use can trigger the activation of microglia and astrocytes in the brain, driving their function to a neuroinflammatory response and inducing the production of pro-inflammatory biomarkers (128, 129). Several of these molecules can cross the blood-brain barrier, promoting immune activation in peripheral tissues. The persistence of this pro-inflammatory feedback loop between the brain and periphery can lead to reduced neuroplasticity, structural and functional changes in key neural circuits underlying cognitive and emotional processing, leading to executive dysfunction, physical disability, and a higher risk of SUD relapse (130) (**Figure 3**).

Consistent with this, prior work supports the relationship between SUDs and peripheral markers of pro-inflammatory biological processes. For example, individuals with AUD exhibit an activation pattern characteristic of a pro-inflammatory response that persists even after alcohol withdrawal (131–133). Cocaine abstinence was also associated with higher levels of pro-inflammatory cytokines (IL-2, IL-6, and IL-17) in a sex-specific manner (134). Importantly, higher IL-6 levels have been correlated with worse EF performance (135). In individuals with opioid use disorder, higher levels of pro-inflammatory cytokines (IL-1β, IL-6) were associated with worse episodic memory performance and worse treatment response to methadone (136, 137). A similar pattern of increased pro-inflammatory activation is also observed in patients with cannabis use disorder (138).

Inflammasome activation is frequently reported in different neuropsychiatric conditions (139–141), where it co-occurs with cognitive dysfunction (142–148). However, the relationship between inflammasome activation and cognitive impairment among adults with SUDs remains understudied. The overarching goals of P3 are to investigate pro-inflammatory changes and inflammasome activation as a mechanism associated with executive dysfunction in SUDs.

### Objectives

The primary objective of P3 is to investigate the impact of inflammasome activation on EF domains in SUDs. Its secondary objective is to investigate the impact of inflammasome activation on structural and functional brain parameters in SUDs (integration with P2).

#### H1

Higher inflammasome activation (i.e., high expression of NALP3) will be associated with: (a) worse inhibition (b) lower working memory updating, and (c) reduced set shifting ability.

#### H2

Higher inflammasome activation will be associated with: (a) lower gray matter in the prefrontal cortex; (b) reduced activation of the default brain network; (c) less connectivity in neural circuits underlying EFs.

### Methods and planned analyses

#### Blood collection, sample preparation, and storage

P3 draws on the same participant pool recruited for P1. Blood is collected into two EDTA (8 cc) and one citrate (4.5 cc) tubes. After processing, the plasma and buffy coat aliquots are immediately stored at -80°C until analysis. Blood samples and extracted DNA are banked for potential future integrative analyses (see Project 7).

#### Laboratory analysis for plasma biomarkers

We will measure the phosphorylated and non-phosphorylated proteins isoform levels of the NALP3 complex using Western Blot. The ratio between non-phosphorylated and phosphorylated isoforms of NALP3 will be used as a proxy measure of the activation of the inflammasome pathway. Additionally, we will measure the plasma levels of the pro-inflammatory cytokines IL-1β, IL-18, and proCaspase-1 using a multiplex immunoassay (bead-based multiparametric assay).

The immunoassays will be analyzed using the Luminex xMAP technology^®^ (Luminex 200). The assay has very low cross-reactivity, and this technology has a higher sensitivity to detect very low concentrations of different analytes. The intra- and inter-assay coefficient of variance for the assay is below 10% for most of the analytes measured.

#### Proposed analysis

To test the association of the biomarkers with the three EF domains and neuroimaging outcomes, we will use Pearson correlation and linear multiple regression models. The linear models can be expanded to include potential covariates, if necessary. The total sample size of 400 subjects will be sufficiently powered to detect small effect sizes of correlation coefficients of 0.11 (α=0.05, statistical power of 80%) to 0.14 (α=0.05, statistical power of 95%).

## Project 4: to assess the role of repetitive transcranial magnetic stimulation (rTMS) on aberrant EF in the context of major depressive disorder in adult outpatients seeking treatment for AUD (NCT06299787)

### Background

Identifying specific neural and molecular mechanisms associated with EF deficits (P2 and P3) may guide future neuromodulatory and pharmacological treatments seeking to boost EF in adults with diverse SUDs and complex clinical presentations. To illustrate the potential utility of brain-based therapies targeting EF, Project 4 (P4) will conduct a clinical trial evaluating the efficacy of neuromodulation on EF in a complex subpopulation of CDiA participants presenting with AUD and Major Depressive Disorder (MDD).

Repetitive Transcranial Magnetic Stimulation (rTMS) of the PFC is FDA-approved for the treatment of MDD and a large body of evidence supports its safety and antidepressant efficacy (149–151). Many cognitive and psychological processes stemming from the PFC, are disrupted in both MDD and AUD (152), which are frequently comorbid (153, 154). Theta Burst Stimulation (TBS) is an efficient form of rTMS associated with both antidepressant effects (155) and alterations in plasticity in the cortex (156). Importantly, TBS takes about 1/6^th^ the time of standard rTMS, translating to a higher impact and consequently more efficient treatment at a population level.

Understanding the mechanisms underlying treatment response to rTMS is crucial to future therapeutic development. Previous studies have demonstrated that both rTMS and TBS target neurophysiological markers of GABA-receptor-mediated inhibition and this mechanism may be related to MDD treatment response (157–160). Although rTMS has shown promise for the treatment of SUDs (E.g (161), there have not been specific neurophysiological investigations into EFs and depressive symptomatology in the context of MDD comorbid with AUD. To address this gap, P4 will conduct a clinical trial evaluating the effects of bilateral TBS on EF in comorbid AUD and MDD. P4 will also investigate the correlation among rTMS treatment, indices of GABAergic inhibition and individual domains of EF (inhibition, updating, and set shifting). To further investigate therapeutic mechanisms, the P4 will incorporate an investigation of theta-gamma coupling (TGC), a neurophysiological index of PFC activity (172), during an N-back working memory task before and after rTMS treatment.

### Objectives

The primary objective of P4 is to conduct a double-blind randomized pilot study, consisting of two arms (bilateral TBS and sham) to investigate the effect of bilateral TBS on deficits in EF in the context of comorbid AUD and MDD. We will also measure alterations in TMS-EEG-indexed GABA receptor mediated inhibition and DLPFC TGC as potential mechanisms mediating therapeutic effects. We will also assess the direct associations between improvement in EF domains after bilateral TBS, with improvement in MDD symptoms.

P4 will test six interrelated hypotheses (H1-H6). Specifically, we hypothesize that, compared with sham TBS, 4 weeks of daily bilateral DLPFC TBS will lead to:

#### H1

Greater improvement in EF task performance;

#### H2

Greater proportion of individuals remaining abstinent from substance use;

#### H3

Greater amelioration of depressive symptoms (as indexed by decrease in HRSD-17 score) and suicidal ideation (as indexed by a decrease in Columbia-Suicide Severity Rating Scale score);

#### H4

Increase in GABA-receptor-mediated inhibition indices;

#### H5

Increase in TGC proportional to improvements in EF;

#### H6 (exploratory)

Improvement in all EF domains (inhibition, updating, and set shifting) in conjunction with improvement in MDD symptoms (ie. more amelioration of MDD symptoms will be associated with improved EF).

### Methods and planned analyses

A total of 40 participants will be recruited from the CDiA pooled sample within 6 to 12 weeks of the baseline assessment for P1. Participants will be randomized in a 1:1 ratio to one of two different rTMS arms. The first arm will include bilateral TBS, applied as cTBS over the right DLPFC followed by iTBS applied over the left DLPFC. The second arm will be sham TBS. All participants will be asked to participate in 4 weeks of treatment, 5 days/week (i.e. weekdays). TMS-EEG will occur at treatment initiation (week 0) and treatment end (week 4). Baseline and post-treatment clinical measures will be administered to characterize each AUD patient. TBS will be administered using the MagPro X100 stimulator equipped with a Cool-B70 coil and Qooler fluid-cooling device (MagVenture, Farum, Denmark) positioned under MRI guidance using the Brainsight neuronavigation system. With a sample size of 20 participants per group and assumed large effect sizes, we will have 80% of statistical power to detect a significant difference between the active treatment arm and sham at a significance level of 0.05.

### Randomization and blinding

We will randomize participants based on a stratified randomization scheme using a permuted block method with a random number generator, in fixed random sizes. We will use an R30 or X100 with a cool A/P B70-type coil (Magventure Inc.) to ensure blinding of both patient and technician. As such, the clinician, researcher, patient and technician will all be blinded. For both active and sham stimulation, the coil is positioned under MRI guidance using real-time neuronavigation, thus providing technician and participant with visual feedback throughout stimulation.

### TBS treatment procedure

TBS will be delivered to the DLPFC bilaterally, with neuronavigation determined through structural MRI. The structural MRI utilized to derive the brain target will have been completed as part of P2. The coil position selection will start by identifying MNI152 stereotaxic coordinates (x, y, z) of (–38, +44, +26) and (26, 38, 44) in the left and right DLPFC, respectively, which we have shown to be effective targets for rTMS in alleviating symptoms of MDD (162). Bilateral TBS will be delivered as follows: R-DLPFC cTBS: 40s uninterrupted bursts (triplet 50 Hz bursts, repeated at 5 Hz, 40s on, 600 pulses total) followed immediately by L-DLPFC iTBS: (triplet 50 Hz bursts, repeated at 5 Hz, 2s on and 8s off, 600 pulses total). For intensity of stimulation we will use 120% RMT. Our work in over 200 people stimulating PFC suggests that 120% RMT is the maximum that should be used for negligible risk of seizure and other serious adverse effects beyond the expected scalp pain and headache during early treatment sessions (163, 164).

### Participants

Patients will be included if they meet general inclusion criteria for CDiA and in addition, if they:

(1) have a DSM–5 diagnosis of AUD based on the Mini-International Neuropsychiatric Interview (MINI (165) (2) do not exhibit problematic use of any substances (excluding nicotine and caffeine), including alcohol, for >1 month; (3) screened positive for an MDE based on the MINI without psychotic symptoms (4) are agreeable to keeping their current antidepressant medications and medications for AUD constant during the study; (5) are reliably taking SUD agonist therapies if appropriate and managed by their clinical team; (6) are able to adhere to the study schedule (7) meet the TMS safety criteria (166).

Participants are excluded if they meet general exclusion criteria for CDiA, and in addition, if they: (1) have a concomitant major unstable medical illness or any significant neurological disorder; (2) are pregnant or intend to get pregnant during the study; (3) have failed a course of ECT, due to the lower likelihood of response to rTMS; (4) have an intracranial implant (e.g., aneurysm clips, shunts, cochlear implants) or any other metal object within or near the head, excluding the mouth, that cannot be safely removed; (5) require a benzodiazepine with a dose equivalent to lorazepam 2 mg/day or higher (167).

Participants will be discontinued from the study if they cannot safely continue the study based on any of the following criteria: (1) experience clinically significant worsening of suicidality that requires an involuntary inpatient hospitalization; (2) develop clinically significant hypomanic or manic symptoms; (3) relapse into problematic substance use in the month prior to rTMS treatment or during the rTMS treatment; (4) miss four rTMS treatments (i.e., 20%); or (5) withdraw consent.

If relapse into substance use is suspected during rTMS treatment, for safety purposes (e.g., to manage seizure risk), a physician will be called to assess the participant prior to the initiation of rTMS treatment for the day or as soon as substance use is suspected. Investigations (e.g., urine drug screen or blood draw) will be ordered at the physician’s discretion, with the consent of the participant. The decision to continue with or discontinue rTMS treatment for that day or going forward will be at the discretion of the assessing physician and the participant’s SUD clinical team.

### Clinical assessments

Participants will be initially screened with the MINI and C-SSRS. Interested and potentially eligible participants will complete a subsequent interview to address all criteria outlined above to confirm eligibility. The 17-item Hamilton Rating Scale for Depression (HRSD-17) (168) will be our tertiary clinical outcome measure. The tertiary clinical outcome criteria will be a decrease in HRSD-17 score at treatment end compared to baseline (150, 162). The HRSD-17 will be performed at baseline, weekly and at the end of the rTMS treatment course. The Columbia-Suicide Severity Rating Scale (C-SSRS) will be used to evaluate suicidality. This reliable and valid scale has been used in randomized clinical trials and is able to predict completed suicide (169). The C-SSRS has been reported as an effective measure for diagnosis and treatment across several diagnoses (170–172) with high internal consistency, and interrater reliability (172–176). The C-SSRS will be performed at baseline, weekly and at the end of the rTMS treatment course. TMS Adult Safety Screen (TASS) (166) will be used at recruitment and baseline to ensure that participants are safe to participate in rTMS treatment trials. To enhance integration with other projects, we will re-administer the EF cognitive assessments within one week prior to the start of rTMS and at the end of the 4-week course of rTMS.

### Neurophysiological indices of GABA receptor mediated inhibition in the DLPFC

To evaluate GABA receptor mediated inhibition indices in the DLPFC, TMS will be administered to the left DLPFC using two Magstim-200 stimulators (Magstim Company Ltd., UK) connected via a Bistim module and electrophysiological data will be collected using dedicated hardware and software (Neuroscan, Compumedics, USA). Each TMS session will include the establishment of the individual threshold for stimulation, followed by GABA receptor mediated inhibition paradigms in the DLPFC according to our published methods (160). Recordings will be acquired through a 64-channel EEG. GABA receptor mediated inhibition indices from the DLPFC will be the dependent variables of interest and derived according to published recommendations (160, 177). Our neurophysiological measures have been established in several of our previous reports and have a high test-retest reliability (i.e., ICC > than 0.9) (178, 179). Data analysis will take place using semi-automated methods developed and validated by our group (160, 178). TMS-EEG will be conducted prior to the start of the course of rTMS and within 48 hours after the last rTMS treatment.

To evaluate TGC, participants will undergo an N-back task (2-back condition) during a 10-minute EEG recording at baseline and after the treatment course as part of the TMS-EEG sessions. EEG signals of TGC are recorded using DC and a low pass filter of 100 Hz at 20-kHz sampling rate, and data processed offline using MATLAB and EEGLAB toolbox, following previously published protocols (180, 181). *Theta-Gamma Coupling*: The measure of TGC is indexed by the modulation index (MI), calculated for each electrode, followed by an average across the right and left frontal electrodes. The MI for all target trials is analyzed as a weighted average based on the number of correct and incorrect responses, as previously described (180, 181).

Finally, we will aggregate data from other CDiA projects (including imaging, genetics, inflammatory models, preclinical biomarkers and more) to further examine the role of GABA receptor mediated inhibition in the cognitive dysfunction found in SUD. Specific baseline cross-analyses will be performed between EF tasks, imaging variables, inflammatory markers and TMS-EEG indices of GABA receptor mediated inhibition.

## Project 5: a preclinical study of the effects of alcohol on EF and the impact of novel therapeutic interventions

### Background

Projects 1-3 will provide insight into the behavioral, neural and molecular underpinnings of EF deficits in SUDs. However, these studies are by design correlational, limiting our ability to establish causal relationships among putatively mechanistic factors. In contrast, P4 will test causal contributions of neuromodulation to EF improvement but is limited to a treatment modality with a demonstrated safety profile. In contrast, Project 5 (P5) will seek to causally establish specific molecular mechanisms and create a platform for testing novel pharmacological treatments targeting EF. To accomplish this, P5 will employ a reverse-translational approach using rats to examine EF-like deficits along the same three dimensions measured in P1-P4. To evaluate causal contributions to EF-like function, P5 will experimentally manipulate similar biological systems to those evaluated correlationally throughout the rest of the CDiA Program.

Preclinical research on substance administration has played a critical role in understanding the nature of substance-related cognitive dysfunction. However, studies have typically focused on single cognitive domains, and relatively little is known about the dynamic trajectories of executive dysfunction [i.e. emergence, dependence and potential reversal (182, 183)]. To address these gaps and complement the human studies in CDiA, P5 will measure in rats how EF-like performance in three domains is affected by chronic alcohol consumption (Objective 1), potential reversal via pharmacological intervention (Objective 2), and identification of peripheral and central biomarkers for translation to humans (Objective 3).

This investigation will target specific biological pathways implicated in EF deficits in AUD, and investigated correlationally across the rest of CDiA. Specifically, P5 will focus on GABAergic neurotransmission, the inflammasome system, the noradrenergic system, and the opioid system. The GABAergic system plays a critical role in cognitive functions (184), and will be investigated as a therapeutic mediator of neuromodulation treatment in P4. To complement findings from P4, P5 will enhance GABA activity pharmacologically during withdrawal in rats to investigate its impact on EFs. Similarly, the activity of the inflammasome, implicated in brain disorders (185) and alcohol consumption (186), will be measured in P3 for its translational biomarker value. To complement and extend P3’s correlational findings, P5 will block inflammasome activity pharmacologically (via MCC950), to assess its impact on EFs. The integrity of the noradrenergic system, to be measured through NM-MRI in P2, plays a critical modulatory role in AUDs (187), with complex effects depending on activity at various receptor subtypes (α1 versus α2). P5 will target the α2-noradrenergic receptor, using guanfacine, an α2-receptor agonist that demonstrated efficacy at reducing alcohol consumption in rats (188) and is used for the treatment of attention-deficit/hyperactivity disorder (189).

Finally, naltrexone is a prescribed medication used to manage alcohol dependence, especially consumption, although it appears to have reduced efficacy in certain patients, depending on gender and genetic differences (190). Naltrexone is predicted to be prescribed in a significant proportion of P1 participants. Its effects on EFs are not fully characterized and tend to vary from study to study (191, 192). Here, we propose to systematically investigate the relationship between its known clinical efficacy on consumption and potential improvement of EFs in rats, providing a clinically-relevant comparison to the other interventions.

### Objectives

The primary objective of Project 5 (P5) will be to characterize the impact of alcohol on three cognitive domains in rats, identify the impact of novel therapeutic approaches, and delineate novel biomarkers predictive of cognitive deficit emergence or treatment efficacy. We will test the following hypotheses:

#### H1

Alcohol withdrawal will increase working memory deficits, impulsivity and cognitive flexibility deficits in adult rats.

#### H2

Cognitive dysfunctions observed will be linked to changes in central biomarker of synaptic functions, changes in various neurotransmitter related activity and peripheral signs of neuroinflammation.

#### H3a

Pharmacological interventions targeting the GABAergic system will contribute to better working memory function, cognitive flexibility and reduced impulsivity during withdrawal.

#### H3b

Pharmacological interventions targeting the noradrenergic system will contribute to better cognitive functions and reduced alcohol seeking behaviors.

#### H3c

Pharmacological interventions targeting the inflammasome will reduce signs of neuroinflammation, indirectly contributing to better cognitive health.

This project will also generate a sample repository (brain, blood, plasma) for investigation and discovery of peripheral and central biomarkers indexing putative mechanisms involved in AUD and executive dysfunction, that could be used for follow-up biomarker analyses.

### Methods and planned analyses

**Figure 4:**
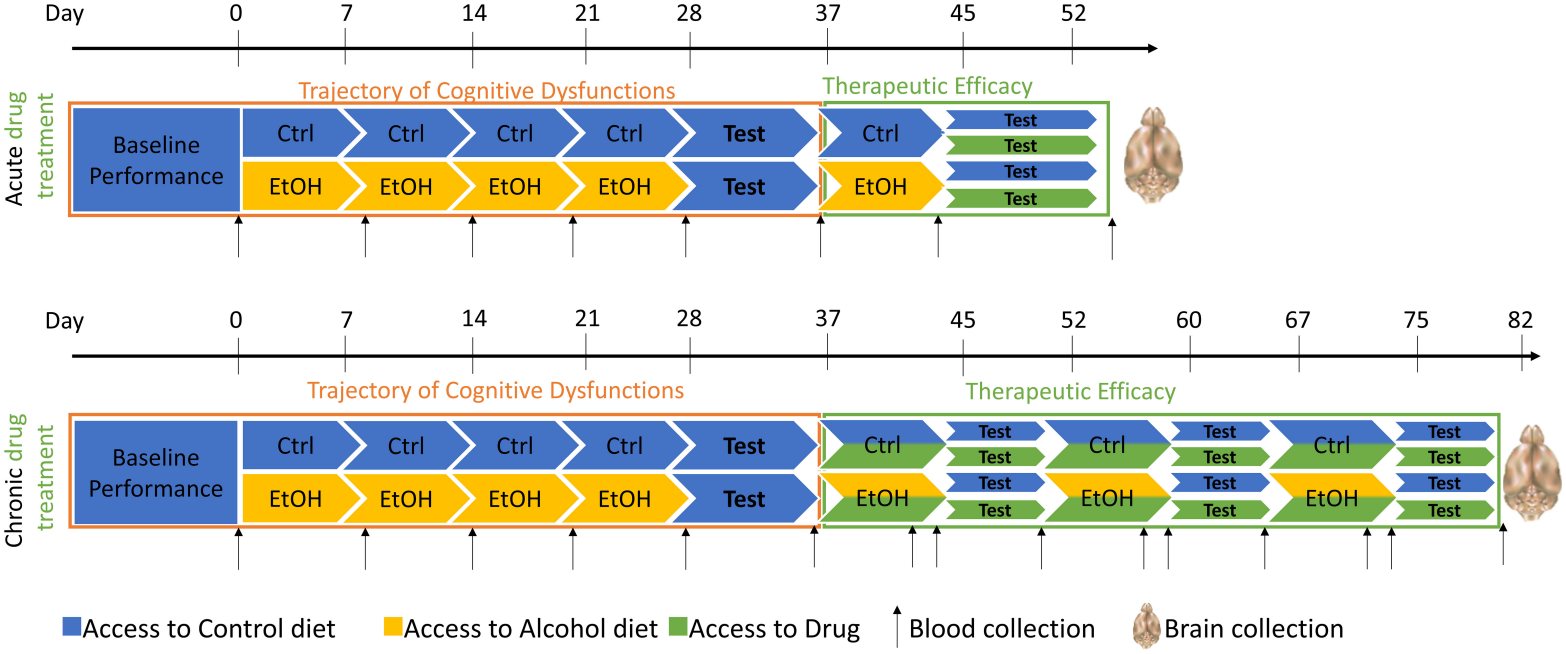
Design of the experiment for cohort of 48 rats. Drugs proposed for this study will be Naltrexone, a5PAM or MC9950. Animals will be tested on different cognitive tasks, depending on their group affiliation (Inhibition/Working Memory or Set-shifting). Blood will be collected on a regular basis to control for the blood alcohol level, and brains will be collected at the end of the study for downstream analysis. Ctrl, control; EtOH, ethanol.

Male and female Long Evans rats, 3 months old at study initiation, will be utilized in this study. Rats will be habituated to consume a liquid diet (Nestlé^®^ Chocolate Protein Boost) in which ethanol can be diluted. After baseline assessment of EFs, half of each cohort will be exposed to a diet supplemented with 10% w/v alcohol 5 days per week. Removal on 2 consecutive days per week will induce a withdrawal period. Such cycle will be repeated 4 times, with assessment of cognitive functions during withdrawal after 4 cycles (**Figure 4**; top panel, orange box).

#### Measures

Three domains of EF (inhibition, working memory, set-shifting) will be assessed to match assessment in P1. Inhibition will be measured using the 5-Choice Serial Reaction Time Task (5-CSRTT). This test is conducted in operant conditioning boxes with 5 apertures featuring integrated light stimuli (193), and a reward dispenser on the opposite wall. Rats are trained to respond to a stimulus among the 5 apertures, earning a reward on a fixed intertrial interval. Lengthening this interval challenges rats to wait longer before responding, with premature responses during the intertrial interval serving as a reliable measure of response inhibition (or impulsivity). In alcohol-exposed rats, premature responses are expected to increase, especially when the intertrial interval is extended.

Working memory will be assessed using a T-maze spontaneous alternation task (194). After 2 days of habituation, rats explore the maze starting in the maze’s initial arm and then choosing between the two goal arms. The chosen arm is closed off, and the rat returns to the start arm for the next trial. This process is repeated for seven trials. Rats naturally alternate between arms, relying on working memory for novel environment exploration. Alcohol-exposed rats tested during withdrawal are expected to show diminished working memory functions.

Set-shifting will be evaluated following Floresco et al.’s protocol (195), in which rats undergo initial training in standard operant conditioning chambers with a house light, two retractable levers, and stimulus lights. Training involves a fixed ratio 1 schedule on each lever, reaching a criterion of 50 rewards or 30 minutes per lever. The second phase introduces a 90-trial session where rats must press a lever within 10 seconds to receive a reward. Visual cue discrimination training follows, with stimulus lights above the levers. Rats respond within 10 seconds to the lit lever for a sucrose reward, while responses on the non-lit lever are punished. Criterion: 10 consecutive correct responses for 30 trials over 4 days. After visual discrimination is learned, rats are tested in a “shift to response discrimination” task, where the rewarded lever is consistent, irrespective of the light stimulus. The number of trials needed to learn this new set serves as a proxy for set-shifting. Rules are modified after each testing session to ensure continual adaptation. Due to the complexity of the inhibition and set-shifting task, these cannot be run in the same animals. Therefore, different animals will be used to run inhibition and working memory vs set-shifting.

Following the assessment of alcohol-induced cognitive function changes, interventions will commence (Objective 2). Treatments involve intraperitoneal administration of drugs targeting α5-GABAA receptors (GL-II-73), the inflammasome (MCC950), the α2-noradrenergic receptor (guanfacine), and the opioid system (naltrexone). Initially, acute dosing will be explored using a Latin square design to test multiple doses (**Table 3**). Conducting multiple doses in the same animal necessitates several testing sessions, and all employed tests, such as the Y maze and 5-CSRTT, are stable and unaffected by practice and repetition (193, 196–198). The set-shifting task (195) can be adapted to establish new rules at each testing session, enabling regular assessment of set-shifting abilities in the same animals while mitigating the impact of practice on repeated testing.

**Table 3:** Low, medium and high doses for each drug tested in Project 5.

Mg/kg	Low dose	Medium dose	High dose
Naltrexone	0.1	0.3	1
α5PAM	3	10	30
MCC950	1	3	10

Blood samples will be drawn weekly and 24 hours after discontinuation of the liquid diet (**Figure 4**) to verify 1) blood alcohol levels (BALs) exceeding 100mg% during exposure and 2) the absence of alcohol 24 hours after cessation when behavioral testing begins. BALs will be assessed using an ANALOX machine. Serum samples will also be utilized for measuring peripheral inflammasome biomarkers. Inflammasome activity will be determined by the phosphorylated/non-phosphorylated NALP3 ratio in peripheral blood mononuclear cells, along with investigating inflammatory markers (NF-KB, ERK, p38MAPK) and cytokines (IL-1β, IL-18, Caspase-1). Luminex xMAP technology^®^ will be employed for biomarker analyses.

After behavioral assessments, rats will be euthanized, and brains dissected for immunofluorescence staining and molecular analyses on regions of interest (prefrontal cortex, VTA, nucleus accumbens and hippocampus). Rostro-caudal coronal sections will be stored at -20°C for neuroinflammation, noradrenergic, and GABAergic marker analysis. RNA and proteins will be extracted from homogenized tissue, allowing for cDNA conversion, quantitative PCR, and Western Blot analyses. The markers examined include, but are not limited to, SST, PV, α5-GABRA, α1 GABRA, BDNF, and other GABAergic markers. Similarly, qPCR, immunostaining, and Western Blot analyses against sterile inflammation biomarkers (NALP3, NF-KB, ERK, p38MAPK, IL-1β, IL-18, Caspase-1) will validate the correlation of peripheral and central biomarkers in a region-specific manner. Investigation of changes in noradrenergic system markers (NET, α1 receptor, α2 receptor) will complement NM-MRI work outlined in P2.

The blood and brain samples collected will form a repository for subsequent biomarker analyses related to executive dysfunction in AUD. As Objective 3 establishes this repository, the analysis of these samples (peripheral or central) will be influenced by findings from other projects. Consequently, additional exploratory investigations of biomarkers will be conducted in these samples when other projects identify relevant pathways or markers in the clinical population.

#### Proposed analyses

Behavioral data will be examined to assess the impact of alcohol exposure on EFs, utilizing ANOVA (or relevant non-parametric alternatives) followed by Tukey’s *post hoc* comparisons for statistical significance. In the alcohol-exposed groups, it is possible to observe subgroups of animals with higher susceptibility to alcohol than the others, suggesting existence of high-responders and low-responders.

For data collected weekly, data will be analyzed with repeated measures to identify animals that are more than 50% of the time over the mean, and animals that are more than 50% of the time under the mean to segregate high-responders and low-responders respectively. If such separation does not exist consistently, then analyses will focus on overall alcohol effect. For the second objective involving drug interactions, separate 2-way ANOVA with diet and treatment as factors will be applied, followed by Dunnett’s *post-hoc* comparisons (198). In Objective #3, blood sample data will be analyzed using Student’s t-test (Objective #1) or 2-way ANOVA (Objective #2) with diet and treatment as factors, followed by Dunnett’s *post-hoc* test. Brain analyses with qPCR and Western Blot will use 2-way ANOVA with diet and treatment as factors, followed by Dunnett’s *post-hoc* comparisons. Pearson or Spearman analyses will evaluate correlations between executive dysfunction and peripheral/central biomarker levels, with p-values adjusted for multiple comparisons using the Bonferroni method due to the potential high number of pairwise analyses.

## Project 6: to assess links between EF and treatment seeking for addiction and healthcare utilization and costs

### Background

P1-P5 will address psychological and biological mechanisms underlying EF and their implications for functional outcomes in SUDs. However, they will not directly evaluate public health impacts and individual experiences with treatment seeking. To complement these mechanistic insights and link them to potential policy implications, Project 6 (P6) will assess the links between domains of EF, treatment seeking and healthcare utilization.

Among individuals with a SUD, EF can affect retention (199–201) and engagement in treatment as well as treatment outcomes (25, 202), including the ability to achieve and maintain abstinence (199–201, 203). Given that SUDs are strongly associated with health care utilization (27), it is also likely that EF, which impacts the success of SUD treatment, is a key factor affecting the extent of health care utilization. In particular, among those with EF deficits, a diminished ability to achieve abstinence or low-risk alcohol use may lead to a higher use of emergency services for the acute consequences associated with SUDs (204). This could result in increased health care costs (205), which is problematic when existing resources dedicated to emergency services are limited (205, 206). Furthermore, a diminished ability to achieve abstinence or low-risk substance use may also lead to a higher use of non-emergency health care services, including primary care visits and hospitalizations, due to acute and chronic health effects associated with substance use (207, 208).

Furthermore, previous research has shown that EF is a factor in engagement in SUD treatment, including more treatment episodes and longer durations of abstinence among those without EF deficits (203). EF deficits lead to worse recognition of problem use (209–211). Therefore, people with EF deficits are more likely to view their symptoms as temporary or not serious, and consequently do not seek treatment (26). These factors may also affect whether patients stay in treatment or remain abstinent after treatment. Accordingly, it is likely that EF deficits affect treatment seeking behaviors, experiences, and barriers among people with SUDs.

### Objectives

P6 has two objectives. The first is to qualitatively assess the potential impact of EF on treatment-seeking behaviors, experiences, and barriers, specifically among individuals with AUD. The second objective is to quantitatively assess the association of EF with health care utilization among individuals with SUD, and to estimate the costs of this utilization.

#### H1

EF deficits will negatively impact treatment seeking and the treatment process as expressed by participants in treatment for AUD.

#### H2

Emergency and non-emergency health care utilization and related costs will be higher among participants in treatment for SUD with more severe EF deficits than among those with less severe EF deficits.

### Methods and planned analyses

#### Objective 1 (qualitative component)

A semi-structured, one-on-one 90-minute interview with a subset of participants recruited from the pooled CDiA sample will be conducted by a research coordinator trained in qualitative methods including interviewing and analysis, shortly after their baseline data are collected. Given differences in treatment pathways and trajectories and their potential impacts on treatment experiences, this analysis will be limited to participants with AUD. Interview questions will be related to treatment-seeking behaviors and EF. Participants will be asked about their experiences seeking help for substance use, perceived facilitators and barriers to treatment, perceptions and expectations regarding treatment, past health care utilization, and questions related to how EF (e.g., memory, attention, impulsivity, etc.) may have played a potential role in their treatment-seeking behavior and experiences. The interview will include probes to encourage participants to reflect on reasons for seeking treatment, including experiences with and perceptions of stigma. Participants will be asked about their social identities (e.g., age, sex and gender, ethnicities, etc.) and their EF, and whether or to what extent these factors have impacted their decision to seek treatment or their ability to benefit from and remain engaged in treatment. Additionally, in the absence of stigma being explicitly discussed by participants, stigma-related responses will be coded at the time of data analysis to interpretively identify the latent impact and role that stigma may have played on participants’ willingness to seek treatment.

This study will undertake a thematic analysis of the data obtained, examining and identifying common themes that emerge related to the perceived impact of EF on treatment-seeking behaviors, experiences, and barriers across the sample. We expect that thematic data saturation will be met with a sample size of approximately 30 participants.

All interview transcripts will be manually reviewed by a member of the research team, and an initial codebook will be prepared based on preliminary analyses. All transcripts will be coded for the initial set of codebook themes using NVivo software. As we anticipate both inductive and deductive themes to emerge from the data, the codebook will be subject to further development and revision based on ongoing analyses and discussion of emergent themes among members of the research team. Data analysis will be finalized once we have met data saturation, and no new themes emerge. Final results will be informed by common themes that emerged and were expressed by multiple participants. To increase data analysis credibility, a secondary independent coder will code a sub-sample of transcripts using the coding framework to assess inter-coder reliability, and any discrepancies between codes or themes will be discussed.

Subsequent to the interview, participants will be asked to complete the Behavior Rating Inventory for Executive Functioning in Adults (BRIEF-A), a standardized validated assessment that captures self-reported perceptions of an individual’s EFs and self-regulation (212). The results of the BRIEF-A assessment tool will be used to better understand the participants’ executive functioning from their perspective, which will further inform our qualitative analysis.

#### Objective 2 (quantitative component)

A health systems evaluation will be conducted to assess the association between EF (as measured in P1) and health care utilization, as well as the costs of this utilization. For this analysis, the database constructed in P1 will be linked with data on health care utilization and the associated costs obtained from the Institute for Clinical Evaluative Sciences (ICES). ICES will provide data on the use of publicly funded health services in Ontario. Participant data will be linked through ICES to the Hospital Discharge Abstract Database (DAD), National Ambulatory Care Reporting System (NACRS), Continuing Care Reporting System (CCRS), Ontario Health Insurance Plan Claims Database (OHIP), and Ontario Drug Benefit Claims (ODB) database. Records-level ICES data linkages will be performed using each participant’s unique Ontario Health Insurance Program health card number; the resulting database will be stripped of all direct personal identifiers and each entry will be assigned a confidential code number. All data linkages will be performed by an ICES data analyst. A subanalysis will be performed stratifying by conditions fully attributable and those not fully attributable to SUD.

Analyses will focus on the occurrence of service utilization, stratified by the type of service (i.e., non-emergency services and emergency services). The occurrence of health care service use among the cohort (recurrent time-to-event data) will be analyzed using multiple models, including covariates across multi-disciplinary domains. These models will include the Andersen and Gill model (i.e., generalizes the Cox model), Prentice, Williams and Peterson (PWP) model, marginal means/rates model, and frailty model (i.e., random effects model) (213–216). The final model to be used will be chosen based upon which of the previously used models best describes the data and which model assumptions are upheld. Model assumptions include the occurrence of an event being dependent upon the prior number of events during the follow-up period (PWP), there being no time-dependent covariates (marginal means/rates model), and the occurrence of an event cannot be explained by observed covariates alone (frailty model). Each statistical model will include covariates for the domains of EF, demographics, substance use, and health service utilization prior to study participation.

## Project 7: to identify subtypes of individuals seeking addiction treatment using cross-disciplinary data types from all projects to and map the biopsychosocial all-cause drivers of cognitive dysfunction in adult outpatients seeking treatment for SUD

### Background

P1-P6 will address targeted hypothesis-driven questions related to EF deficits and health outcomes in SUDs. To complement these hypothesis-driven efforts, Project 7 (P7) will integrate data types collected across all other projects to identify novel data-driven participant profiles and evaluate their utility in predicting outcomes over time.

In SUDs, heterogeneity is the rule rather than the exception (217). This heterogeneity is largely driven by extremely high rates of comorbid psychiatric conditions (218–220) and compounded challenges in the diagnostic process, as emergent cognitive syndromes may be temporary, drug induced, or chronic (221, 222). Even when considering the same SUD, symptoms and prognoses vary wildly between patients due to complex combinations of life experience (223), biology (224), and socio-demographic factors (225, 226). Given this heterogeneity, it is likely that there exist subpopulations, or subtypes (227–229), of individuals with similar diagnostic classifications but different underlying disease mechanisms and degrees of cognitive impairment. Due to these mechanistic differences, interventions aimed at improving deficits in EF (230) would not be expected to work equally well in different subtypes, meaning their identification is a priority for future applications of precision medicine in SUDs.

Attempts to define subgroups within and across diagnoses with relatively homogeneous symptom profiles and outcomes have met with limited success; for example, a five-biotype model of alcohol dependence has been proposed using latent class modeling of family history, age at onset, DSM-IV criteria, and data on comorbid illnesses (231). Others have proposed four- and two-group subtyping schemes based on psychiatric co-morbidity, family history, psychopathy, temperament, and other clinical health measures (232). Despite the strong links between symptom dimensions and executive dysfunction (see P1), subtypes have not been formally evaluated for differences in domains of inhibition, set shifting, or working memory updating, leaving a major gap in the field. To their detriment, most efforts have focused on relatively few domains of interest, not examined changes in cognitive performance (specifically EF) over time, and yielded varying, unstable results. Therefore, it is important to approach the problem from a data-inclusive perspective (233). Using insights from unimodal investigations, such as those proposed in the preceding CDiA projects, it is also possible to assemble optimal parsimonious predictive models at the individual level which could aid in screening efforts and clinical decision making.

### Objectives

Within Project 7 (P7), we propose to integrate data collected from each of the proceeding projects – including use of insights from animal experiments in Project 5 – to (1) perform multi-modal subtyping on SUD individuals, (2) evaluate subtypes against known biological risk profiles for cognitive dysfunction and psychiatric illness (such as exploring polygenic risk scores) and other longitudinal outcomes (e.g. TMS treatment outcome, specified in P4), and (3) use machine learning approaches to optimize predictors of cognitive dysfunction over time and to understand the structure of modifiable risk and resilience factors that may present opportunities for intervention. Within this broader framework, P7 will test the following specific hypotheses:

#### H1

Multi-modal unsupervised and semi-supervised clustering of SUD patients at baseline will reveal transdiagnostic subtypes of SUD with unique risk factor profiles and levels of EF.

#### H2

Subtypes from Aim 1 will be predictive of changes in EF over one year of follow-up and individuals with evidence for subgroup transition will experience different treatment response rates.

#### H3

Multivariate predictive models of changes in EF over time, incorporating the available multi-modal feature space of CDiA, will offer better performance than models based on individual modalities.

### Methods and planned analyses

#### Cross-sectional data-driven subtyping

This will involve using subject clustering algorithms to identify relatively homogeneous subgroups of patients. Primary measurements from each of P1-6 will be used as input for clustering (see **Table 4** for domain definitions). The similarity network fusion (SNF) method will then be used to combine more fine-detailed, high-dimensional input variables across multiple modalities. To address the key overarching theme of this proposal, subgroups based on EF domains (including and excluding other measures, as in P1 objectives) will be of particular interest, and will be tested for distributional differences in primary prognostic outcomes of functional recovery and sustained health. The semi-supervised association-boosted SNF (abSNF) method will be used to identify subgroups with most significance to EF performance at baseline. Internal validation of group membership will be performed using bootstrap procedures. Socio-demographic, educational, and life experiential characteristics of each subgroup will be described and placed within the context of existing cross-sectional work.

**Table 4:** Summary of assessment domains and measurements across study projects.

Project	Domain (instrument/source)	Measures
1	Clinical (DART, suicidal ideation) + Substance use (TFLB, AUDIT, DUDIT, SDS)	Psychiatric diagnoses, comorbidity, suicidal ideation, childhood adversity, medication, treatment engagement
		Recent and lifetime history of (poly)substance use
		Age of onset, average yearly use, heaviest period of use
	Cognition (CNS-VS, executive function tasks, other behavioral tasks)	Global cognitive function
		Executive function (Inhibition, updating, set shifting) deficits and biases
		Risk and reward behavior
	Functional impairment and quality of life (WHOQOL-BREF, WHODAS)	Physical, psychological, social, and environmental
		Cognition, mobility, self-care, getting along, life activities, and participation
2	Neuroimaging (3T MRI)	Task-based and resting state fMRI
		T1-weighted structural MRI
		Neuromelanin-sensitive MRI of SN/VTA and LC
		Diffusion MRI
3	Genomics	Assay-based genome-wide genotypes
		Assay-based genome-wide methylation profiles
	Plasma biomarkers	Inflammasome proteins (NF-KB, ERK, and p38MAPK, NALP3)
		Pro-inflammatory cytokines (IL-1β, IL-18, and ProCaspase-1)
4	Intervention (4 week TMS) clinical outcomes	Suicidal ideation (SSI)
		Depressive symptoms (HRSD-17)
	Intervention electrophysiological outcomes	Electroencephalogram (EEG)-derived GABA receptor mediated inhibition

This table does not list every measurement from every project and core, though it provides an overview of the types of measurements belonging to each general domain analyzed in P7. P5 is absent, as measures taken from rodent models will not be directly included as features in human sample statistical modeling.

#### Longitudinal stability of baseline subgroups and subgroup transitions

Within- and across-data type clustering will be performed independently at multiple time points and evaluate the frequencies of individual-level membership transitions. This aim is similar in nature to clustering approaches used by P1 (H1d and H1e). However, the methods are adapted to account for the increased diversity and dimensionality of input data that will be used to identify latent subgroups. In P1, latent profile analysis and then latent growth curve modelling will be used to identify clusters of participants, and to determine if individual trajectories of substance use and functional outcomes are predicted by baseline EFs, respectively. P7 will extend the foundational work of P1 by examining patient trajectories based on data collected across projects 1-6 and determined using different, network-based methodologies. Critically, we will also identify socio-demographic, educational, and life experiential factors determining inconsistency in group membership over time. Subtypes based on integrative data analyses will be evaluated against more traditional clinical subtypes (such as those from P1) to determine if distributions of key demographics and outcomes are modified by the inclusion of multi-disciplinary input features.

#### Supervised machine learning for prognostication

We will apply machine learning methods on features identified by each project across domains to build optimally predictive models of: (1) EF, (2) social function, (3) suicidality and self-harm, and (4) healthcare system burden (234). Modelling will proceed iteratively, using processes developed by our group (235–237).

## Discussion

There is widespread recognition of the need for integrative and longitudinal research incorporating cognitive, biological, and clinical measures of vulnerability and resilience in SUDs. Within the CDiA Program structure, detailed clinical and cognitive characteristics of a treatment-seeking sample will be integrated with biological markers closely linked to both cognitive dysfunction and SUDs, permitting the identification of critical mechanisms and therapeutic targets. Linkages to healthcare utilization and innovative whole-person modeling approaches will further take this initiative from “neuron-to-neighborhood” and foster integrative and translational research consistent with recent calls to action in this field.

A main strength and innovation of the CDiA Program consists of its translational team and unique collaborative research approach on executive dysfunction for functional recovery and sustained health in outpatients seeking treatment for SUDs (**Figure 1**). Other conceptual advantages of the Program are the adoption of an individual differences approach, appreciation for patient complexity and heterogeneity, and the recognition that transformation of care can only occur when combining expertise from clinical neuroscience, clinical care, clinical research, and epidemiology/health science research perspectives. As depicted in **Figure 1**, P1-4 are closely linked investigations with a focus on collecting deep data in complex patients seeking treatment for SUDs; these human subjects research projects emphasize ecological validity and will occur in parallel with Project 5, which offers the opportunity to investigate associations between EFs, proposed biological underpinnings, and associated pharmacological therapeutics in a highly controlled and efficient design. P6 and P7 extend the generativity of this work, with a focus on healthcare utilization and costs, as well as lived experience perspectives, and the application of highly sophisticated, advanced computational modeling to integrate data across all other projects. To further demonstrate the intended integrated approach across projects, we provide here selected examples of potential for individual projects benefiting and feeding back on strategy, approach and deliverables of other projects (see **Table 5**).

**Table 5:** Cross-Disciplinary collaboration across projects.

Clinical and cognitive measures collected within P1 will be linked to neuroimaging biomarkers collected within P2, peripheral biomarkers collected within P3, and healthcare utilization and costs indexed in P6.
Clinical and cognitive measures collected within P1 will further characterize participants with elevated suicidality who receive rTMS as part of P4, and contribute to the whole person modeling planned for P7.
Executive function tasks included in both P1 and P5 were carefully chosen to ensure consistency with current models of executive function and with each other. P1 will also track pharmacological treatments, and exploratory analyses of the effects of naltrexone on executive functioning in P1 will be informed by P5 preclinical models of the effects of naltrexone and other medications on executive functioning.
Neuroimaging measures collected within P2 will be mapped onto peripheral molecular markers assessed in P3
The dopamine and norepinephrine systems, whose integrity will be indexed with NM-MRI, are conserved across species and can be manipulated in preclinical models, hence creating opportunities for collaboration between P2 and P5
Innovative therapeutic approaches targeting key mechanisms involved in AUDs and executive dysfunction, such as GABAergic deficits (P4) and inflammasome activation (P3), will be tested to characterize their efficacy at reducing domains of alcohol-related executive dysfunction in P5.
P5 will provide an experimental assay for AUD-related executive dysfunction and a repository of biological samples for future proof-of-concept studies aimed at novel biological pathways discovered in this project (i.e. P4)
Both P4 and P5 target the GABA system, permitting the evaluation of innovative treatment alternatives that may show promise in those with alcohol use difficulties and associated cognitive dysfunction
P5 targets the inflammasome using innovative compounds as well and will synergize with P3 biomarker studies and P7 integration studies
The clinical, imaging, and peripheral markers within P1, P2 and P3 can be utilized to predict health care utilization and the costs of this utilization within P6
P6 will investigate which of the associations of executive function deficits with SUD treatment experiences and healthcare utilization differ across those with and without pervasive suicidality, and thus will inform and be informed by P4
P6 will investigate whether the associations of executive function deficits and health care utilization and costs differ among those who used naltrexone versus those who did not, and will thus inform and be informed by P5
P7 is the end-cap of the analytical pipeline for the proposed study and the key point of integration across preceding projects. Knowledge generated in P1-6 will be essential for determining which of the measured variables across domains will be most informative in statistical modeling.

Our inclusive approach to participant recruitment will allow us to recruit a large sample representative of the complex clinical populations typically seen in tertiary care settings. We recognize this will also pose analytical challenges related to sample heterogeneity. Our reliance on multi-level measures of transdiagnostic relevance (e.g., EF, functioning, neural circuit function, inflammatory markers) and our state-of-the-art data integrative approaches outlined in P7 place the CDiA Program in a strong position to harness this heterogeneity to improve understanding of “real-world” presentations of SUDs and their cognitive features. This can in turn accelerate impactful discovery science, clinical translation, and societal/policy changes needed to improve outcomes in this population.

It is anticipated that following the recruitment of this initial cohort, subsequent research will investigate the impact of specific interventions (20, 238) on impaired EFs and functional outcomes. Thus, it is the vision to use these results to support an “experimental medicine” approach aimed at remediating cognitive dysfunction. Peripheral and imaging biomarkers identified in our initial cohort will permit such a nuanced investigation of mediators of clinical outcomes, and in conjunction with preclinical investigations, will inform the development of novel therapeutics (239, 240). Although both psychosocial (e.g., cognitive training) and biological (e.g., pharmacotherapy, noninvasive brain stimulation) interventions are forecasted, the first planned intervention will investigate the efficacy of rTMS on outcomes in SUDs. However, participants in the broader CDiA program are expected to be enrolled in a variety of treatment programs across CAMH and beyond. Thus, although Projects 1-3 of the broader CDiA Program are not centered on a specific clinical trial, they are well-positioned to capture symptom changes and recovery across a variety of therapeutic contexts over time. Given the intended generativity of the program, we do anticipate that the current work will give rise to new, larger and potentially more comprehensive treatment trials directed by initial insight from CDiA.

### Limitations

This Research Program is not without limitations. First and foremost, the sample in P1-P3 is by design expected to be heterogeneous in terms of specific substance use profiles and comorbid diagnoses. At the same time, given the different eligibility requirements and diversity of methods (e.g., MRI safety requirements in P2), not all Projects’ samples will be representative of the full CDiA cohort. Notably, P4 and P5 represent smaller experimental studies targeting specific biological mechanisms and use narrower clinical phenotypes. Second, we recognize that the 12-hour abstinence window used in P1-P3 may not account for all residual effects of recently used alcohol or other substances on cognition. To account for this residual variability, all analyses will control for data on recency of use, collected through various means such as self-report and urine drug screens (**Table 1**).

### Implications and future directions

Limitations aside, the CDiA Program is uniquely positioned to bridge expertise across multiple complementary scientific domains and perspectives that have typically remained siloed in SUDs research to date. Future initiatives may wish to expand this model to other clinical populations, treatment modalities, and experimental systems. Future efforts could also explore alternative qualitative analytical methods (e.g., interpretative phenomenological analysis (241)), as well as further participatory action research and designs. For instance, online photovoice is a recently developed innovative qualitative research method (242–246). Online photovoice gives participants the opportunity to express their own experience with as little manipulation as possible, compared to some traditional quantitative methods (244–246). This may be particularly relevant for the topic of cognitive dysfunction among individuals with SUD and represents a promising direction for future work. Community-based participatory action research (247) and culturally responsive small-group work (248) may further support the needs of this population in an inclusive and equitable way in future studies.

Limitations notwithstanding, with its diverse and complementary projects and a shared focus on EF, the CDiA Program is poised to make groundbreaking strides in understanding the heterogeneity of SUDs. The Program also holds promise to identify novel behavioral and neurobiological mechanisms, along with key experiential factors, that could inspire innovative treatments and policies to improve SUD outcomes.

## CDiA program study group

CDiA Program Study Group consists of LQ, ES, DF, SL, TP, YN, AR, JW, EV, DV, Isabelle Boileau, Nikki Bozinoff, DB, Christopher Bowie, Paul Fletcher, PG, AH, Colin Hawco, Yarissa Herman, Pamela Kaduri, BF, OM, LQ, Jürgen Rehm, KS, MS, VT, WW and SW.

## Footnotes

1 Throughout this protocol paper, we use the term “substance use disorder” or “SUD” broadly to include alcohol use disorder and other substance use disorders, unless noted otherwise.
